# Biosynthesis of Polyhydroalkanoates Doped with Silver Nanoparticles Using *Pseudomonas putida* and *Pseudomonas aeruginosa* for Antibacterial Polymer Applications

**DOI:** 10.3390/ijms25168996

**Published:** 2024-08-19

**Authors:** Carmen Liliana Cruz-Romero, Abraham Ulises Chávez-Ramírez, Cyntia R. Flores-Juárez, Noé Arjona, Alejandra Álvarez-López, Laura del Bosque Plata, Vanessa Vallejo-Becerra, Juan de Dios Galindo-de-la-Rosa

**Affiliations:** 1 Facultad de Ingeniería, División de Investigación y Posgrado, Centro Universitario Cerro de las Campanas, Universidad Autónoma de Querétaro, Querétaro, Qro. C.P. 76010, Mexico; lilicanar@gmail.com (C.L.C.-R.); alejandra.alvarez@uaq.mx (A.Á.-L.); 2 Centro de Investigación y Desarrollo Tecnológico en Electroquímica, Pedro Escobedo, Qro. C.P. 76703, Mexico; achavez@cideteq.mx (A.U.C.-R.); wvelazquez@cideteq.mx (N.A.); 3 División Industrial Área de Nanotecnología, Universidad Tecnológica de Querétaro, Querétaro, Qro. C.P. 76148, Mexico; cyntia.flores@uteq.edu.mx; 4 Instituto Nacional de Medicina Genómica, Cuidad de México, CDMX. C.P. 14610, Mexico; ldelbosque@inmegen.gob.mx

**Keywords:** polyhydroxyalkanoates, *Pseudonomas putida*, *Pseudonomas aureginosa*, silver nanoparticles

## Abstract

In this study, the biosynthesis of polyhydroxyalkanoates (PHAs) was carried out using *Pseudomonas* putida and *Pseudomonas aeruginosa*. These PHAs were produced using reagent-grade glycerol and crude glycerol as the carbon sources. The objective was to compare the production of PHAs and to functionalize these polymers with silver nanoparticles to provide antibacterial properties for potential biomedical applications. The findings from the physical and chemical analyses confirmed the successful synthesis and extraction of PHAs, achieving comparable yields using both crude glycerol and reagent-grade glycerol as carbon sources across both strains. Approximately 16% higher PHAs production was obtained using *Pseudomonas putida* compared to *Pseudomonas aeruginosa*, and no significant difference was observed in the production rate of PHAs between the two carbon sources used, which means that crude glycerol could be utilized even though it has more impurities. Notably, PHAs functionalized with silver nanoparticles showed improved antibacterial effectiveness, especially those derived from reagent-grade glycerol and the *Pseudomonas aeruginosa* strain.

## 1. Introduction

Polyhydroxyalkanoates (PHAs) are aliphatic polyesters produced by bacteria from diverse habitats and ecological niches, including renewable sources, organic acids, fossil resources, and waste materials [1]. Due to their properties, such as biocompatibility and slow hydrolytic degradation, PHAs have been increasingly used in packaging and biomedical fields [2]. In the medical sector, the Food and Drug Administration has approved their use as a biomaterial for absorbable sutures [3]. Large-scale production of biodegradable plastics is primarily limited by the relative expense of the substrate. Substrate costs represent approximately 40% of the total PHA production costs [4]. This is why finding an unconventional substrate source, such as waste derived from biodiesel that can pose an environmental risk, could reduce the production costs of this type of polymer [5]. Biodiesel is a fuel made up of monoalkyl esters (methyl, ethyl, or propyl) of long-chain fatty acids, which are sourced from vegetable oils or animal fats [6]. Its worth as a fuel has been acknowledged since the 19th century; the base-catalyzed transesterification of vegetable oil was performed four decades before the first diesel engine began operation [7]. Glycerol is the main residual from biodiesel production, with about 10 kg of crude glycerol generated per 100 kg of biodiesel. The substantial volume of this by-product has spurred efforts to discover sustainable and valuable applications for glycerol, underscoring its critical role in biodiesel manufacturing and beyond. Researchers are actively exploring innovative ways to utilize glycerol efficiently, aiming to enhance the economic and environmental sustainability of biodiesel production processes [8].

The surplus of crude glycerol generated in the biofuel industry has caused a decline in glycerol prices, and in recent years, many biodiesel production plants have even considered it waste (with associated disposal costs). As a result of this global situation, glycerol has emerged as a highly attractive substrate for bacterial fermentations [9]. Transforming crude glycerol into value-added products has become a critical necessity to boost the sustainability and economic viability of the biofuel industry. This conversion not only helps in managing the by-product efficiently but also adds financial and environmental value to the biofuel production process [10], and both chemical and biological methods have been investigated to transform glycerol into more valuable products using suitable microorganisms. Crude glycerol mainly consists of free fatty acids and fatty acid methyl esters, which can selectively enhance the utilization of carbon substrates for bacterial growth and PHAs biosynthesis. Produced in large amounts, crude glycerol has been identified as an attractive raw material for bacterial production of high-value products such as PHAs [11]. 

*Pseudomonas* are recognized for their ability to metabolize more than 80 organic carbon compounds, including sugars, amino acids, carboxylic acids, simple aromatic compounds, paraffinic hydrocarbons, and terpenes. Numerous studies have explored the utilization of glycerol by *Pseudomonas* species. These investigations have delved into biochemical and genetic aspects, uncovering unique metabolic pathways, genetic regulatory mechanisms, and physiological adaptations specific to *Pseudomonas*, distinguishing them from other bacterial species. These insights contribute to a deeper understanding of how *Pseudomonas* effectively processes glycerol, potentially enhancing its application in various biotechnological and industrial contexts [9,12]. *Pseudomonas* species are potentially selected for PHAs production due to their possession of enzymes such as PHAs synthase (PhaC1 and PhaC2Ps). These enzymes demonstrate a wide substrate specificity, accommodating monomers containing carbon chains ranging from 4 to 12 atoms in length [13]. For the production of PHAs by *Pseudomonas*, it utilizes the ß-oxidation pathway of fatty acids. Initially, fatty acids undergo conversion into acyl-CoA thioesters, which are subsequently oxidized via ß-oxidation. This process involves sequential steps through trans-2-enoyl-CoA and (S)-3-hydroxyacyl-CoA intermediates, ultimately forming 3-ketoacyl-CoA. This 3-ketoacyl-CoA is then cleaved by -ketothiolase, leading to the production of acetyl-CoA and an acyl-CoA molecule with a reduced carbon chain length compared to its precursor entering the initial cycle [14]. This process repeats in successive cycles until the initial acyl-CoA is fully converted to acetyl-CoA. Enzymes like enoyl-CoA hydratase (encoded by phaJ) and 3-ketoacyl-CoA reductase (encoded by fabG) are pivotal in transforming intermediates of fatty acid ß-oxidation into appropriate substrates, particularly (R)-3-Hydroxyacyl-CoA, which is subsequently polymerized by PHAs synthase [3]. Genetic data were gathered to reconstruct the glycerol metabolism pathway in *Pseudomonas aeruginosa* and *Pseudomonas putida*, revealing similarities to the aerobic glycerol processing pathway found in *Escherichia coli*. The series of reactions, facilitated by ATP-dependent GlpK kinase and ubiquinol-dependent GlpD dehydrogenase, converts glycerol into dihydroxyacetone. This compound serves as a gateway into central carbon metabolism, playing a crucial role in substrate transport and utilization [15]. 

The addition of some antibacterial agents to PHAs produced from *Pseudomonas* becomes a necessity for their effective application in the biomedical sector. Creating antibacterial surfaces that effectively prevent the formation and growth of biofilms represents a promising strategy to combat bacterial infections by impeding their establishment and transmission. These surfaces not only hinder initial bacterial adhesion but also disrupt biofilm maturation, thereby mitigating the risk of infection spread and enhancing overall hygiene standards in various environments [16]. The most common nanocomposites used as antibacterial films are based on silver, which is well known for its strong toxicity to a wide range of microorganisms with high temperature stability and low volatility [17,18,19]. Silver nanoparticles have attracted considerable attention in response to the increasing prevalence of antibiotic-resistant microbial pathogens. These nanoparticles hold promise for diverse biomedical applications, encompassing wound dressings, coatings, and tissue scaffolds, where their antimicrobial properties can contribute to enhanced therapeutic outcomes and infection control [17,20]. In this research, the bacteria *Pseudomonas aeruginosa* and *Pseudomonas putida* were evaluated for the production of PHAs using reagent-grade glycerol and crude glycerol from the biodiesel industry. The polymers obtained were doped with silver nanoparticles, characterized, and their antibacterial activity evaluated. 

## 2. Results 

### 2.1. Evaluation of PHAs Synthesis in the Different Strains of Pseudomonas putida and Pseudomonas aeruginosa 

**Figure 1:**
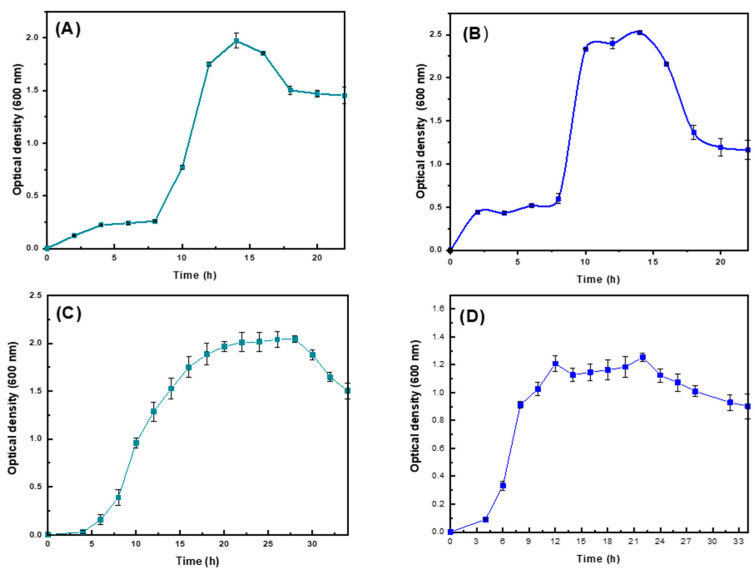
The bacterial growth rate of strains *Pseudomonas aeruginosa* (**A**,**B**) and *Pseudomonas putida* (**C**,**D**) was measured in a basal medium of minimal salts modified with reagent-grade glycerol (**A**,**C**) and crude glycerol (**B**,**D**) as the sole carbon source.

Figure 1 shows the growth curves for the *Pseudomonas putida* and *Pseudomonas aeruginosa* strains. The exponential phase appears in both cases at around 10 h. It was observed that the *Pseudomonas putida* strain exhibits a faster adaptation to the culture medium and a greater capacity to survive compared to *Pseudomonas aeruginosa*, as its growth phase is prolonged and cell death is delayed, suggesting [21] that it utilizes the produced organic matter more efficiently as a source of material and energy, a characteristic reported in the literature [22,23]. 

The growth of both *Pseudomonas putida* and *Pseudomonas aeruginosa* was assessed using crude glycerol and reagent-grade glycerol as carbon sources at various time intervals. *Pseudomonas aeruginosa* exhibited the fastest growth on crude glycerol in the initial 24 h, with an exponential phase doubling time of 0.508 h and reaching a higher DO600 value compared to the other experimental conditions. *Pseudomonas aeruginosa* cells cultured in reactive-grade glycerol showed a significant delay phase in the first 10 h compared to the other cells; however, their growth rate increased after 10 h. Its doubling time was 1.0066 h during the exponential phase. Regarding the *Pseudomonas putida* strain, slightly delayed growth was observed compared to *Pseudomonas aeruginosa*, with a doubling time during the exponential phase of 1.7354 h for *Pseudomonas putida* cells cultured in crude glycerol and 4.1806 h for *Pseudomonas putida* cells cultured in reactive-grade glycerol. It is noted that the overall growth behavior of the bacteria does not change significantly when supplementing the medium, thus going through all their known kinetic stages and obtaining PHAs as a secondary metabolite, a typical characteristic reported for *Pseudomonas* [9,24], according to the literature, there are no notable variations in gene expression levels of enzymes purportedly involved in PHAs synthesis when cells are cultivated with various carbon sources like glucose, glycerol, citrate, or lauric acid. Therefore, it is inferred that genes responsible for PHAs synthesis from glycerol in *Pseudomonas*, such as glpF, glpK, and glpD, may similarly influence the process using crude glycerol. 

**Figure 2:**
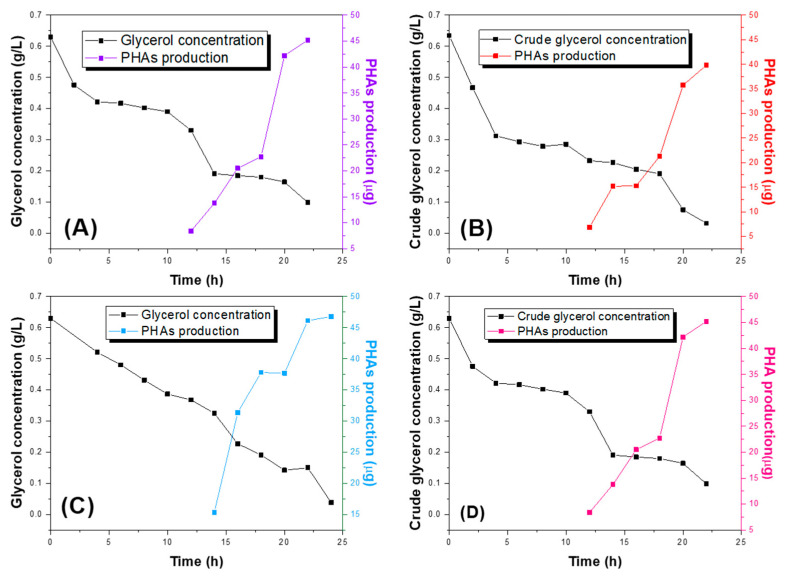
Substrate consumption rate and PHAs production. Substrate consumption rate in black, glycerol (**A**,**C**), crude glycerol (**B**,**D**), and biopolymer production in blue of the strains *Pseudomonas aeruginosa* (**A**,**B**) and *Pseudomonas putida* (**C**,**D**).

Figure 2 depicts the consumption of reagent-grade glycerol and crude glycerol, along with the production of PHAs. The maximum production of PHAs commences around hour 18 in both *Pseudomonas aeruginosa* and *Pseudomonas putida* strains, corresponding to the end of the stationary phase, indicating that biopolymer production is a secondary metabolite. For the *Pseudomonas aeruginosa* strain, after the culture period, 39.82 µg of biopolymer was extracted, with 3.2% of crude glycerol remaining in the medium. In contrast, in the medium enriched with reagent-grade glycerol, 45.21 µg of biopolymer was obtained with 9.8% remaining glycerol. Similarly, for the P. putida strain, 37.39 µg of PHAs were obtained, with 3.0% of crude glycerol present, while in the medium with reagent-grade glycerol, 46.13 µg of polymer was obtained with 14.8% of glycerol remaining. 

Better PHAs production from crude glycerol is observed with the *Pseudomonas aeruginosa* strain, likely due to its higher level of local repeats compared to the genome of the *Pseudomonas putida* strain, which may have played a role in DNA mutations, reflecting the strain’s adaptation to glycerol contaminants. This adaptation resulted in increased tolerance and capacity for storing and converting crude glycerol, even with contaminants, into PHAs. However, the production of biopolymer decreased in both strains compared to that produced from reagent-grade glycerol. 

Although the consumption of crude glycerol leads to the onset of maximum PHAs production earlier compared to reagent-grade glycerol in the *Pseudomonas putida* strain, occurring approximately after 16 h, it does not result in higher biopolymer production or improved performance in using this substrate. 

Table 1 shows the production of PHAs using glycerol and crude glycerol, where it can be seen that there is greater efficiency using *Pseudomonas putida*. The differences in substrate production and assimilation between each bacterial strain are possibly attributed to their central metabolism. For instance, *Pseudomonas aeruginosa* encodes only one copy of the five transporters for gamma-aminobutyric acid (GABA; PP4106, PP2911, PP4756, PP2543, and PP0284), which may be involved in butyric acid uptake, subsequently converting it into polyhydroxyalkanoic acids (bioplastics). On the other hand, *Pseudomonas putida* possesses only two incomplete TRAP family dicarboxylate transporters (PP1167, PP1169), whereas *Pseudomonas aeruginosa* has at least four. Additionally, *Pseudomonas putida* has only one sugar transporter, PTS for fructose (PP0795, PP0792–793). A low amount of PHAs was extracted from both strains and from the two carbon sources, consistent with literature reports [11,25,26,27], approximately 10%. This could be due to various losses in the extraction process, such as during multiple washings. 

### 2.2. TGA of the PHAs Produced 

**Figure 3:**
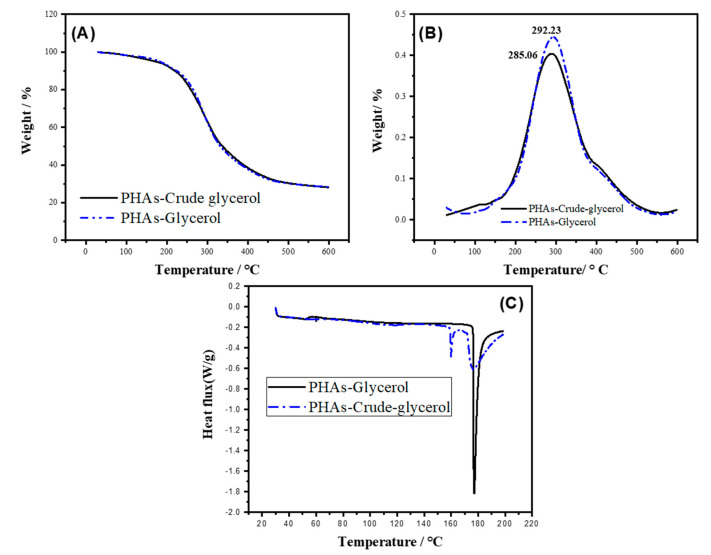
Thermogravimetric analysis and differential thermal analysis for the PHA obtained from *Pseudomonas aeruginosa* strain. TGA for *Pseudomonas aeruginosa* (**A**,**B**) *Pseudomonas putida* (**B**); (**C**) DSC heating curves for the PHAs obtained.

The initial degradation temperature and maximum degradation temperatures of PHAs-glycerol and PHAs-crude-glycerol were calculated from the first derivative of the TGA curves. Additionally, the temperature at a weight loss of 10% and the residue (rPHA600%) were determined from the TGA curves. The results are depicted in Figure 3. 

A peak at around 450 °C on a TGA curve of polyhydroxybutyrate (PHB) could also be attributed to impurities possibly introduced from the carbon source during the synthesis, processing, or handling of PHB. These impurities, including organic contaminants with higher thermal stability than PHB, might degrade at higher temperatures. 

Figure 3C shows the first heating curves of PHAs from crude glycerol and pure glycerol. The PHAs-Glycerol sample exhibits its glass transition temperature at 67.13 °C, while the PHAs-Crude-Glycerol sample has two different glass transition temperatures at 57.33 °C and 159.78 °C. The first transition temperature (57.33 °C) is significantly lower (−10.0 °C) than the first Tg of the PHAs-Crude-glycerol sample, indicating greater flexibility at room temperature. The melting point of PHAs-Glycerol (177.03 °C) is slightly higher than that of PHAs-Crude-Glycerol (176.7 °C); however, the melting point is practically the same for both polymers. 

### 2.3. Silver Nanoparticles Characterization 

**Figure 4:**
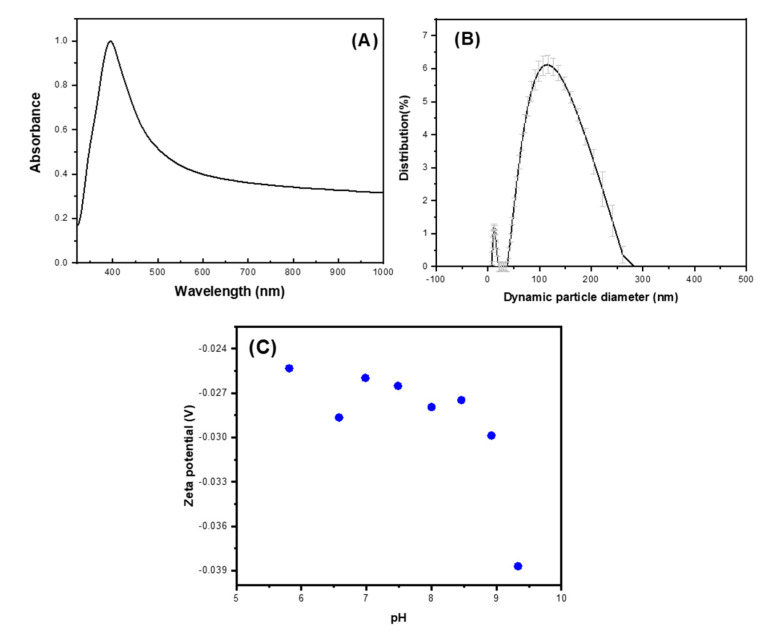
(**A**) UV-vis absorption spectrum of silver nanoparticles; (**B**) Intensity-based particle size distribution of an aqueous suspension of silver nanoparticles obtained from DLS analysis; (**C**) measure- ments as a function of pH for silver nanoparticles suspended in water and acidified with hydrochloric acid solutions.

UV-VIS absorption spectra are recognized for their high sensitivity to silver nanoparticles’ formation, owing to their intense absorption signals arising from surface plasmon resonance. This phenomenon elucidates the collective oscillation of conduction electrons within the metal, providing valuable insights into nanoparticle synthesis and characterization. Understanding these spectra facilitates precise analysis and optimization of silver nanoparticle production processes in various applications [28]. For the characterization of the synthesized silver nanoparticles, UV-vis spectra were recorded. The shift in color from white to yellow signifies the production of silver nanoparticles, evidenced by an absorption peak at 395 nm. The formation of silver nanoparticles was corroborated through the chemical reduction approach. Figure 4A. displays the UV–Vis spectra of the silver nanoparticles within the range of 200–1000 nm. The absorption band in the visible light region (340–550 nm, with a plasmon signal at 400 nm) is characteristic of silver nanoparticles [29]. 

The particle size distribution revealed two populations: the primary population (comprising 90% of the sample) had a mean hydrodynamic diameter of 95 nm, while the secondary population (constituting 10% of the sample) had a mean diameter of 4 nm (Figure 4B). The data’s particle size distribution exhibited a polydispersity index of (PI = 0.2716). 

The aggregation status was assessed by examining the zeta potential, which denotes the electrical potential at the interface between the diffuse ion layer enveloping the particle surface and the surrounding solution. This analysis provides crucial insights into particle stability and dispersion behavior in the solution. The Anton Paar Litesizer 500 (Anton Paar, Graz, Austria) was used to determine the zeta potential of the silver nanoparticles in suspension at different pH levels. It was found that the silver nanoparticles retain a surface charge ranging from −38 mV to −25 mV at pH levels of 9.3 to 5.8, respectively, presenting a relationship similar to that previously reported [30], where the stability of the silver nanoparticles generally decreases as the pH value is reduced, approaching the isoelectric point at pH close to 1.5. According to the literature [30] and the DLVO theory (Derjaguin, Landau, Verwey, and Overbeek) [31], it is suggested that the suspension of silver nanoparticles is stable and does not agglomerate in this pH range due to a dominant repulsive force. This interaction results from both attractive van der Waals forces and repulsive electrostatic forces between particles experiencing Brownian motion. This phenomenon is influenced by Sodium Borohydride, which not only reduces Silver Nitrate but also stabilizes silver nanoparticles through surface adsorption, creating a negative surface charge and generating repulsive forces. These insights guided the determination of the pH range suitable for the physical blend of silver nanoparticles and PHAs. 

### 2.4. Characterizations of PHAs with Silver Nanoparticles 

**Figure 5:**
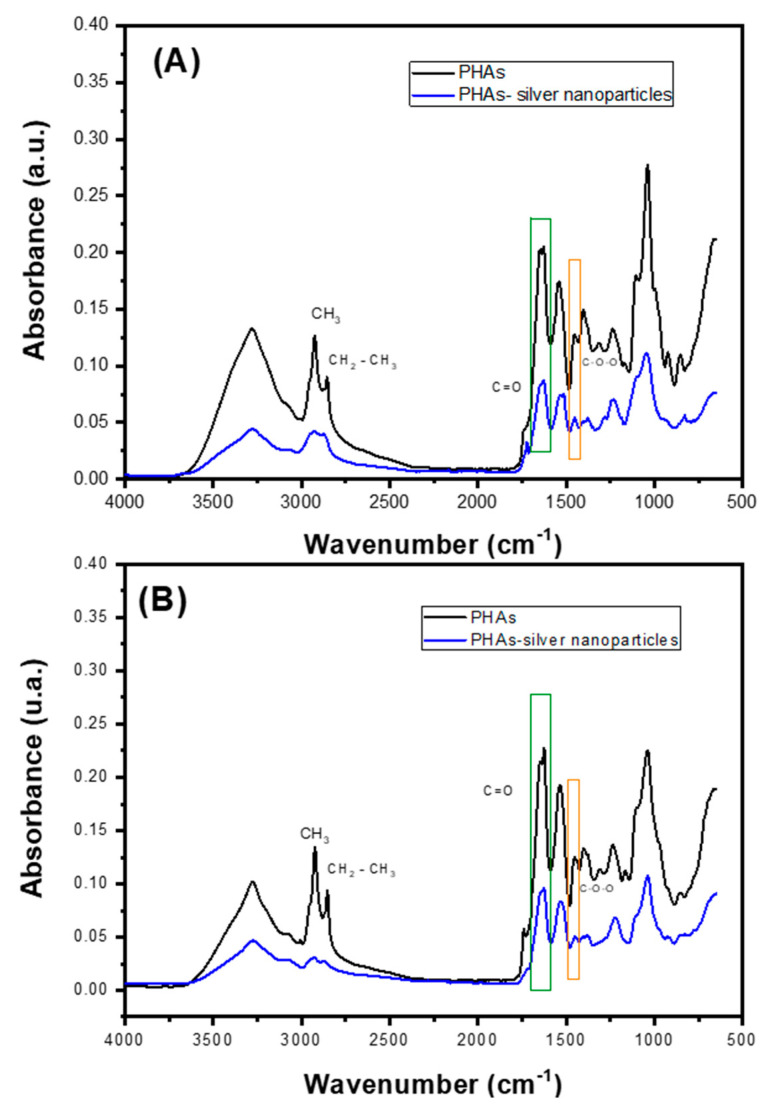
FTIR analysis of PHAs from *Pseudomonas aeruginosa* using (**A**) reagent-grade glycerol and (**B**) crude glycerol.

The extracted PHAs sample underwent non-destructive attenuated total reflectance FT-IR analysis (Figure 5). The infrared absorption observed between 3274 cm^−1^ and 3277 cm^−1^ was attributed to intermolecular hydrogen bonds [32]. The absorption band observed from 2922 cm^−1^ to 2956 cm^−1^ corresponds to the asymmetric C-H stretching vibrations of the methyl groups in the monomeric side chains. Additionally, the absorption band from 2848 cm^−1^ to 2866 cm^−1^ is associated with CH_3_ groups [33]. The absorption band between 1724 cm^−1^ and 1749 cm^−1^ is reported as a PHAs marker band assigned to the stretching vibration of the carbonyl ester bond (C=O) [25,29,33]. The vibrations observed from 1624 cm^−1^ to 1641 cm^−1^ are distinctive markers indicating the presence of poly-3-hydroxybutyrate (P3HB). Additionally, vibrations between 1447 cm^−1^ and 1463 cm^−1^ are attributed to poly(3-hydroxybutyric-co-hydroxyvaleric acid), P(HB-co-HV). The absorptions ranging from 1165 cm^−1^ to 1322 cm^−1^ correspond to the asymmetric C–O–C stretching vibration, while the signal between 1230 cm^−1^ and 1247 cm^−1^ is recognized as characteristic of PHB. These spectral features provide critical insights into the chemical composition and structure of the biopolymer under study [25,29]. A similar spectrum was observed for the PHAs-silver nanoparticle materials compared to the spectrum of each material without silver nanoparticles. A decrease in signal intensity is noticeable after adding silver nanoparticles to each sample, consistent with literature findings that show almost identical spectra for PHAs containing nanoparticles [33,34]. 

**Figure 6:**
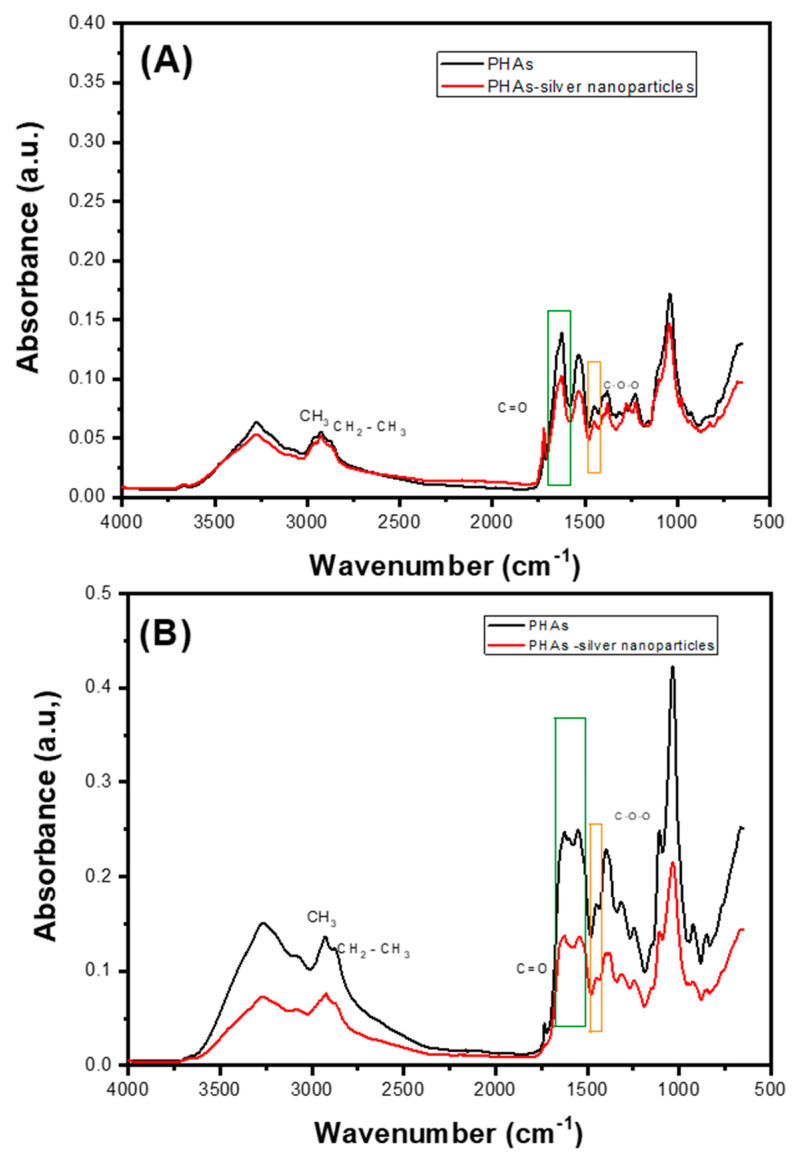
FTIR analysis of PHAs from *Pseudomonas putida* using (**A**) reagent-grade glycerol and (**B**) crude glycerol.

Polymers with siver nanoparticles synthesized from *Pseudomonas aeruginosa* (Figure 6) showed a greater decrease in signal intensity, regardless of the substrate, compared to those from *Pseudomonas putida*. This reduction could indicate a stronger electrostatic interaction between the silver nanoparticles and the PHAs. 

A reduction in the intensity of peaks corresponding to functional groups involved in binding hydroxyl or carbonyl groups in C-H stretching, shifts in the C-H stretching region (2800–3000 ccm^−1^) if there is modification of the hydrocarbon chain, and changes in the carbonyl stretching frequency (around 1700 cm^−1^) might indicate covalent attachment of silver nanoparticles. PHAs have carbonyl groups that could interact with silver, leading to the possible formation of silver-oxygen bonds (Ag-O) if silver nanoparticles interact with ester or hydroxyl groups in PHAs [35]. 

### 2.5. Micrographs of the PHAs-Silver Nanoparticles Nanocomposite 

**Figure 7:**
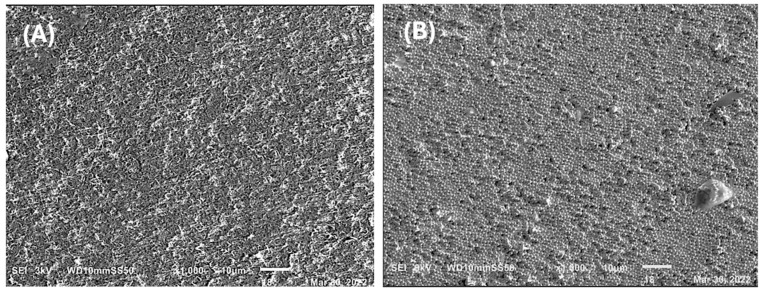
Scanning electron micrographs showing the morphology of films formed from *Pseudomonas aeuriginosa* with reagent-grade glycerol. The control films without silver nanoparticles (**A**) and the films with silver nanoparticles (**B**).

**Figure 8:**
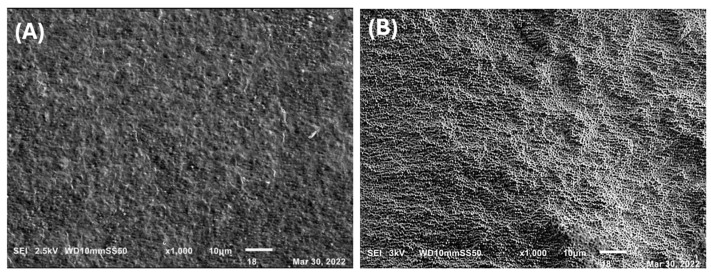
Scanning electron micrographs showing the morphology of films formed from *Pseudomonas putida* with reagent-grade glycerol. The control films without silver nanoparticles (**A**) and the films with silver nanoparticles (**B**).

A study of material morphology was conducted using scanning electron microscopy (SEM), examining each material both with and without 25 ppm of silver nanoparticles. All samples exhibited porous surfaces. Samples from the *Pseudomonas aeruginosa* bacteria showed a more uniform morphology compared to those from *Pseudomonas putida*. Specifically, the PHAs obtained from *Pseudomonas aeruginosa* using reagent-grade glycerol displayed a granulated surface, consistent with literature findings [36] (Figure 7), indicating uniformity, and exhibited a morphology resembling intertwined rods. PHAs obtained from *Pseudomonas putida* using reagent-grade glycerol as a substrate showed less uniformity in morphology compared to those from *Psedomonas aeruginosa* and glycerol. Meanwhile, PHAs from *Pseudomonas putida* and crude glycerol displayed a honeycomb-like morphology (Figure 8). 

The X-ray diffraction pattern of PHAs obtained from the *Psedomonas aeuriginosa* strain and reagent-grade glycerol is included in the Supplementary Information. Morphologically, the PHAs did not exhibit significant changes after the addition of silver nanoparticles, except for an increase in roughness that created valleys and ridges. This roughness is likely due to the interaction between silver nanoparticles and PHAs. A more pronounced formation of valleys and ridges was observed in PHAs obtained from *Pseudomonas aeruginosa* using reagent-grade glycerol as a substrate. 

### 2.6. Antibacterial Evaluation of the Silver Nanoparticles-PHAs Composite 

**Figure 9:**
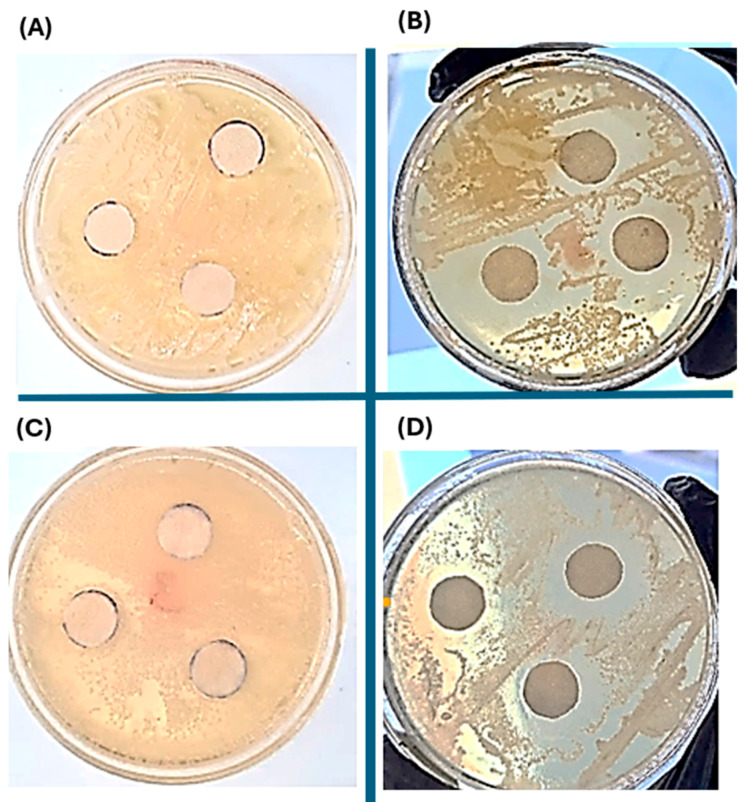
Antibacterial inhibition zone of the discs impregnated with PHAs-silver nanoparticles produced with the *Pseudomonas aureginosa* strain, with two different concentrations of silver nanoparticles: 0.05 mg/kg (**A**,**C**) and 0.5 mg/kg (**B**,**D**). Figures (**A**,**B**) correspond to the PHAs produced from reagent-grade glycerol, while figures (**C,D**), the PHAs formed with crude glycerol.

**Figure 10:**
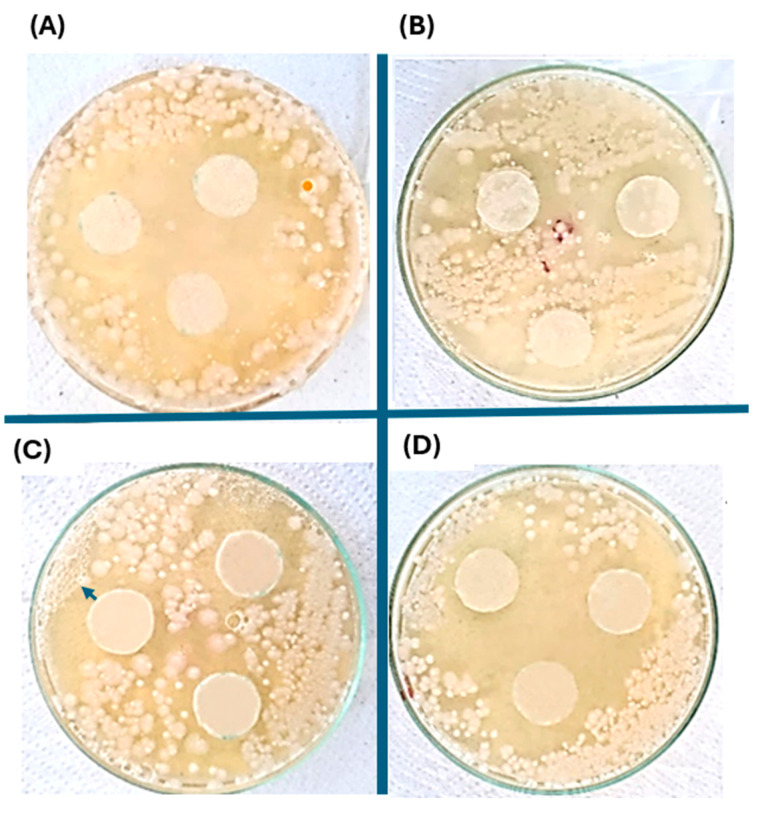
Antibacterial inhibition zone of the discs impregnated with PHAs-silver nanoparticles produced with the *Pseudomonas putida* strain, with two different concentrations of silver nanoparticles, 0.05 mg/kg (**A**,**C**) and 0.5 mg/kg (**B**,**D**). Figures (**A**,**B**) correspond to the PHAs produced from reagent-grade glycerol, while figures (**C**,**D**), the PHAs formed with crude glycerol.

The antibacterial study was conducted using the disk test, employing two different concentrations of silver nanoparticles (0.05 mg/kg and 0.5 mg/kg) for each type of PHAs obtained. The biopolymer derived from the *Pseudomonas aeruginosa* strain exhibits a larger inhibitory diameter compared to that from *Pseudomonas putida*, possibly due to better interaction with silver nanoparticles and greater retention of them. However, it is observed that in all types of biopolymers with silver nanoparticles, antimicrobial activity diffuses radially from the disk through the agar, resulting in a decrease in concentration as it moves away from the disk. Eventually, the concentration of biopolymers with silver nanoparticles in the medium becomes insufficient to inhibit the growth of the *Staphylococcus aureus* strain at a certain distance from the disk Figure 9 and Figure 10. 

It is hypothesized that the structure of the primary cell wall, particularly the glycan chains and peptide branches, could be altered by silver nanoparticles. While there are existing reports on the mechanisms of silver nanoparticles in *Staphylococcus aureus* as a model bacterium for Gram-positive organisms, further investigations are necessary to comprehensively understand their effects on cell wall components and associated biochemical pathways [37], and detailed documentation on the mechanism of action of silver nanoparticles remains limited. Nonetheless, it is proposed that their antibacterial effects may parallel those of conventional antimicrobial agents used in treating bacterial infections. Existing literature identifies three main mechanisms of nanoparticles: initial adherence to the cell membrane, leading to alterations in energetic functions like permeability and respiration. Subsequently, they inhibit bacterial cell wall synthesis in *Staphylococcus aureus*, contributing to their antibacterial activity. Further research is essential to fully elucidate these mechanisms and their potential applications in combating bacterial infections effectively [38]. 

Table 2 shows the comparison of the antibacterial inhibitory zones using a concentration of silver nanoparticles. The antibacterial efficacy of colloidal silver nanoparticles has been shown to be affected by several attributes of the particles, including their size, shape, surface charge, and stability in different environments. These characteristics play crucial roles in determining the effectiveness of silver nanoparticles against microbial pathogens [39]. Smaller nanoparticles, which provide a larger surface area for interaction, are hypothesized to exhibit more potent bactericidal effects compared to larger particles. This observation is partially supported by the findings of dynamic light scattering (DLS) analyses, which revealed that 90% of the sample exhibited a mean hydrodynamic diameter of 95 nm, while 10% of the sample had an average diameter of 4 nm. This suggests that smaller nanoparticles could potentially offer enhanced inhibition of bacterial growth due to their increased surface area-to-volume ratio.

## 3. Materials and Methods

Silver nitrate, sodium borohydride, chloroform, sulfuric acid, Triton X-100, ammonium sulfate, dipotassium phosphate, and magnesium sulfate were purchased from Sigma-Aldrich (Queretaro, Mexico); bacteriological agar, reagent-grade glycerol, and tryptic soy were purchased from Merck (Mexico City, Mexico). The *Pseudomonas aeruginosa* strain was provided by Centro de Investigación y de Estudios Avanzados (CINVESTAV) from its National Collection of Microbial Strains, and the *Pseudomonas putida* strain was donated by the Autonomous University of Queretaro, Mexico. 

### 3.1. Inoculation and Incubation of Bacteria for PHAs Production 

The inoculum for the plate growth of bacterial strains was carried out in a minimal basal saline medium with the following composition: 10 g glucose, 3 g (NH_4_)_2_SO_4_, 3 g K_2_HPO_4_, 0.2 g MgSO_4_, and 15 g bacteriological agar, incubated at 36 °C. Subsequently, a liquid growth culture medium enriched with reactive-grade glycerol and crude glycerol was prepared. This was done by suspending 3 colonies of each strain in different Erlenmeyer flasks, one with 0.63 g/L of reactive-grade glycerol and another with 0.63 g/L of crude glycerol, both containing 100 mL of Tryptic Soy Broth. The incubation process was conducted at 150 rpm and 37 °C for one week. Bacterial growth was evaluated using a UV-visible spectrophotometer. Sampling was conducted at various incubation intervals, with daily monitoring of diverse process parameters aimed at optimizing conditions for PHAs production. These parameters included carbon source utilization (p/p%), biomass yield (g/L), and PHA yield (g/L). The experiments were carried out in triplicate to ensure the reliability and consistency of the results. 

### 3.2. Extraction of PHAs from Microbial Cultures of Pseudomonas aeruginosa and Pseudomonas putida

The method used for biopolymer extraction was solvent extraction, chosen because it does not degrade the polymer [40]. Cells were separated via centrifugation at 10,000 rpm and 4 °C for 15 min. A wash with acetone was performed to remove any organic matter from the cell surface [41]. Subsequently, a second wash was conducted using the anionic surfactant Triton X-100 to permeabilize the cells and extract the organelles [42]. Subsequently, the cells were resuspended in 50 mL of chloroform and incubated on an orbital shaker at 180 rpm, maintained at 30 °C for 24 h to facilitate the extraction of the biopolymer [1]. The chloroform solution containing the precipitate was evaporated, yielding a concentrated solution of PHAs polymers, which was subsequently weighed. 

### 3.3. Quantification of PHAs through the Spectrophotometric Assay for Poly-β-Hydroxybutyric Acid

An estimate of the polymer amount produced was made based on the assumption that the type of PHAs obtained was poly-hydroxybutyric acid, utilizing a gravimetric procedure. This method is based on the fact that this polymer is soluble only in boiling chloroform, ensuring the removal of all contaminants through extraction with other solvents [43]. The method operates on two key principles: firstly, poly-β-hydroxybutyrate is completely converted into crotonic acid through heating in concentrated sulfuric acid, and secondly, the ultraviolet absorption peak of crotonic acid shifts to 235 nm when concentrated sulfuric acid serves as the solvent [44]. UV-visible spectra were obtained using a Thermo Scientific Genesys 10S UV-VIS spectrophotometer, with measurements performed in a 10 mm quartz cell. To conduct the spectrophotometric assay, a sample of the polymer dissolved in chloroform was transferred into a clean test tube for analysis. The chloroform was evaporated, and then 10 mL of concentrated sulfuric acid was added to the residue. The tube was capped and heated for 10 min at 100 °C in a water bath. After cooling, the solution was mixed thoroughly, and a portion was transferred to a silica cell for measuring absorbance at 235 nm against a blank of sulfuric acid. 

### 3.4. Physicochemical Characterization of PHAs 

The detection of the infrared spectrum of isolated PHAs was carried out by dispersive infrared spectroscopy. Infrared spectra were recorded on a Spectrum Two FT-IR spectrophotometer. A thermogravimetry analysis (TGA) and a differential scanning calorimetry (DSC) study were performed as described in the literature [41], in order to determine the thermal stability of the PHAs. The samples were heated at a rate of 10 °C/min from ambient temperature to 600 °C under a nitrogen atmosphere. The decomposition temperature (Td) was recorded. Differential scanning calorimetry was conducted from −80 to 180 °C at a heating rate of 10 °C/min under a nitrogen atmosphere. The following parameters were measured: glass transition temperature (Tg), melting temperature (Tm), crystallization temperature (Tc), heat of fusion (Hm) during melting, and heat of crystallization (Hc). 

### 3.5. Synthesis and Characterization of Silver Nanoparticles 

The synthesis of silver nanoparticles was carried out by the chemical reduction of silver nitrate using sodium borohydride according to the following reaction [45]:

$${\mathrm{AgNO}}_{3}+{{\mathrm{NaBH}}_{4}}^{-}\;\to \;\mathrm{Ag}+\frac{1}{2}H_{2}+\frac{1}{2}B_{2}+{\mathrm{NaNO}}_{3}$$

Four different suspensions of each type of unpurified PHAs and silver nanoparticles were prepared. Synthesis was carried out for three different concentrations of silcer nanoparticles: 0.05 µg/mL, 0.5 µg/mL, and 50 µg/mL. An excess of sodium borohydride was required to reduce silver and stabilize the AgNPs’ form, using a 3:1 ratio of a 2 mM NaBH_4_ solution to a 1 mM AgNO_3_ solution. AgNO_3_ was added dropwise (approximately 1 drop per second) to the previously prepared NaBH_4_ solution in an ice bath on a magnetic stir plate. The complete addition took around 15 min, after which the stirring was stopped, and the stir bar was removed [46]. The analysis was performed using dynamic light scattering (DLS) of a suspension of silver nanoparticles in distilled water at a 1:5 concentration using the Anton Paar Litesizer 500 instrument (Anton Paar, Graz, Austria). The measurements were conducted at 25 °C and were repeated five times to assess sedimentation and solution stability. The aggregation state was determined by analyzing the charge at different pHs in order to determine optimal solution conditions for the physical mixture of the silver nanoparticles with the PHAs. The Anton Paar Litesizer 500 (Anton Paar, Graz, Austria) was used to determine the zeta potential of silver nanoparticles. 

### 3.6. Synthesis of the Nanocomposite (PHAs-Silver Nanoparticles) 

A modified version of the method described by Potter was utilized [47]. The four types of PHAs obtained and extracted (0.002% *w*/*v*) were resuspended in distilled water and subjected to sonication for 3 h. Subsequently, physical mixtures of each type of biopolymer were prepared with three different concentrations of silver nanoparticles, resulting in concentrations of 0.05 mg/kg, 0.5 mg/kg, and 50 mg/kg of silver nanoparticles in PHAs, and they were sonicated for 3 h as well. Finally, they were stored for antibacterial testing. This nanocomposite was modified to create films in order to evaluate the antibacterial effect. The morphology of the nanocomposites was examined by scanning electron microscopy (SEM) with a JSM-6010LA Scanning Electron Microscope (Jeol, Japan). The selected samples were PHAs with a concentration of 0.5 mg/kg silver nanoparticles; they were subsequently dried at 40 °C for 1 h in the oven to obtain films, and for analysis, they were coated with a layer of Au to provide them with conductive properties. 

## 4. Conclusions 

According to the standards used for synthesizing PHAs from crude glycerol and reagent-grade glycerol, variations were observed in the amount of biopolymer obtained. It is suggested that the properties of crude glycerol itself, particularly its contaminants, interfered with the metabolism of both bacterial strains, *Pseudomonas aeruginosa* and *Pseudomonas putida*, leading to a decrease in production by 11.9% and 18.9%, respectively, for PHAs obtained from crude glycerol. However, it is still considered a substrate with potential for PHA production. Possibly due to greater tolerance to contaminants in crude glycerol, the *Pseudomonas aeruginosa* strain achieved a production of 88.1%, while the *Pseudomonas putida* strain reached 81.1%, compared to production from reagent-grade glycerol. 

The polymer obtained from both reagent-grade glycerol and crude glycerol showed very similar thermal and physical properties based on DSC and TGA characterization techniques. They exhibited close values for physical properties such as Tm, Tg, Td, and crystallinity. Regarding silver nanoparticles, their stability, reflected in the zeta potential graph within the pH range of 6 to 10, was −26 mV and −40 mV, respectively. This positive characteristic was reflected in their manipulation to synthesize the silver nanoparticle-PHA composite, indicating the variation in the silver nanoparticle sintering method used. 

There appears to be greater interaction between the biopolymer and silver nanoparticles in the composite formed from reagent-grade glycerol and the *Pseudomonas aeruginosa* strain compared to other composites. This is suggested due to its initial granular morphology before the addition of silver nanoparticles. FTIR analysis of the composite, comparing the spectrum of the biopolymer before and after adding silver nanoparticles, showed greater changes in signal intensities, indicating that silver nanoparticles are likely embedded in the polymer chains, thus reducing vibrations of different functional groups. Additionally, the size obtained from the silver nanoparticles, reported by DLS to be less than 100 nm for most of the population, could facilitate the possible internalization of silver nanoparticles through the polymer chains of the composite. 

## Figures and Tables

**Table 1:** PHAs production according to the substrate used.

	*Pseudomonas aureginosa*	*Pseudonomas putida*
Glycerol	0.084757708 (g/L)	0.099539043 (g/L)
Crude glycerol	0.08499393 (g/L)	0.097921247 (g/L)

**Table 2:** Inhibitory zone values for antibacterial evaluations using 0.5 mg/kg of silver nanoparticles.

	*Pseudomonas aureginosa* Inhibitory Zone Distances (mm)	*Pseudonomas putida* Inhibitory Zone Distances (mm)
Glycerol	11.36	5.49
Crude glycerol	10.53	12.48

## Data Availability

Data are contained within the article and Supplementary Materials.

## Supplementary Materials

The following supporting information can be downloaded at: https://www.mdpi.com/article/10.3390/ijms25168996/s1. Refs. [48,49] are cited in the Supplementary Materials.

## Abbreviations

The following abbreviations are used in this manuscript:

PHAs	Polyhydroxyalkanoates

## References

[1] Rond’ošová S., Legerská B., Chmelová D., Ondrejovič M., Miertuš S. (2022). Optimization of Growth Conditions to Enhance PHA Production by Cupriavidus necátor. Fermentations.

[2] Kalia V.C., Patel S.K., Lee J. (2023). Exploiting Polyhydroxyalkanoates for Biomedical Applications. Polymers.

[3] Rai R. (2010). Biosynthesis of Polyhydroxyalkanoates and Its Medical Applications. Ph.D. Thesis.

[4] Teeka J., Imai T., Cheng X., Reungsang A. (2010). Screening of PHA-Producing Bacteria Using Bio- diesel-Derived Waste Glycerol as a Sole Carbon Source. J. Water Environ..

[5] Rocha-Meneses L., Hari A., Inayat A., Yousef L.A., Alarab S., Abdallah M., Shanableh A., Ghenai C., Shanmugam S., Kikas T. (2023). Recent advances on biodiesel production from waste cooking oil (WCO): A review of reactors, catalysts, and optimization techniques impacting the production. Fuel.

[6] Nisa F. (2023). U Chapter 12—Biofuel: A unique solution for the future energy crisis. Environmental Sustainability of Biofuels.

[7] Henriques R. (1898). Über partielle Verseifung von Ölen und Fetten II. Angew. Chem..

[8] Garlapati V.K., Shankar U., Budhiraja A. (2016). Bioconversion technologies of crude glycerol to value added industrial products. Biotechnol. Rep..

[9] Poblete-Castro I., Wittmann C., Nikel P.I. (2020). Biochemistry, genetics and biotechnology of glycerol utilization in Pseudomonas species. Microb. Biotechnol..

[10] Singh R., Langyan S., Rohtagi B., Darjee S., Khandelwal A., Shrivastava M., Kothari R., Mohan H., Raina S., Kaur J. (2022). Production of biofuels options by contribution of effective and suitable enzymes: Technological developments and challenges. Mater. Sci. Energy Technol..

[11] Wang H., Li H., Lee C., Mat Nanyan N.S., Tay G.S. (2024). A systematic review on utilization of biodiesel-derived crude glycerol in sustainable polymers preparation. Int. J. Biol. Macromol..

[12] Rossini Simões C., Pereira da Silva M.W., Magalhães de Souza R.F., Roja Hacha R., Gutierrez Merma A., Torem M.L., Cianga Silva F.P. (2024). Biosurfactants: An Overview of Their Properties, Production, and Application in Mineral Flotation. Resources.

[13] Tsuge T., Yano K., Imazu S., Numata K., Kikkawa Y., Abe H., Taguchi S. (2005). Biosynthesis of Polyhydroxyalkanoate (PHA) Copolymer from Fructose Using Wild-Type and Laboratory-Evolved PHA Synthases. Macromol. Biosci..

[14] Taguchi K., Tsuge T., Matsumoto K., Nakae S., Taguchi S., Doi Y. (2001). Investigation of metabolic pathways for biopolyester production. RIKEN Rev..

[15] Silby M.W., Winstanley C., Godfrey S.A.C., Levy S.B., Jackson R.W. (2011). Pseudomonas genomes: Diverse and adaptable. FEMS Microbiol. Rev..

[16] Verlinden R.A.J., Hill D.J., Kenward M.A., Williams C.D., Radecka I. (2007). Bacterial synthesis of biodegradable polyhydroxyalka-noates. J. Appl. Microbiol..

[17] Bruna T., Maldonavo-Bravo F., Jara P., Caro N. (2021). Silver Nanoparticles and Their Antibacterial Applications. Int. J. Mol. Sci..

[18] Pulignam I., Appaturi J.N., Parumasivam T., Ahmad A., Sudesh K. (2022). Biomedical Applications of Polyhydroxyalkanoate in Tissue Engineering. Polymers.

[19] Mamata, Agarwal A., Awasthi A., Awasthi K., Dutta A. (2023). Antibacterial activities of GO–Ag nanocomposites with various loading concentrations of Ag nanoparticles. Appl. Phys. A.

[20] Naganthran A., Verasoundarapandian G., Khakid F.E., Masarudin M.J., Zulkharnain A., Nawawi N.M., Karim M., Che Abdullah C.A., Ahmad S.A. (2022). Synthesis characterization and biomedical application of silver nanoparticles. Materials.

[21] Javid H., Nawaz A., Riaz N., Mukhtar H., UI-Haq I., Ahmed Shah K., Khan H., Naqvi S.M., Shakoor S., Rasool A. (2020). Biosynthesis of Polyhydroxyalkanoates (PHAs) by the Valorization of Biomass and 494 Synthetic Waste. Molecules.

[22] Sillema W.D., Cal A.J., Hathwaik U.I., Orts W.J., Lee C.C. (2023). Polyhydroxyalkanoate production in *Pseudomonas putida* from alkanoic acids of varying lengths. PLoS ONE.

[23] Liu H., Chen Y., Zhang Y., Zhao W., Guo H., Wang S., Xia W., Wang S., Liu R., Yang C. (2022). Enhanced production of polyhydroxyalkanoates in *Pseudomonas putida* KT2440 by a combination of genome streamlining and promoter engineering. Int. J. Biol. Macromol..

[24] Spiers A.J., Buckling A. (2000). The causes of Pseudomonas diversity. Microbiology.

[25] Getachew A., Berhanu A., Birhane A. (2016). Production of Sterilized Medium Chain Length Polyhydroxyalkanoates (Smcl- Pha) as a Biofilm to Tissue Engineering Application. J. Tissue Sci. Eng..

[26] Guo W., Duan J., Geng W., Feng J., Wang S., Song C. (2000). Comparison of medium-chain-length polyhydroxyalkanoates synthases from Pseudomonas mendocina NK-01 with the same substrate specificity. Microbiol. Res..

[27] Shamala T.R., Divyashree M.S., Davis R., Kumari K.S.L., Vijayendra S.V.N., Raj B. (2009). Production and characterization of bacterial polyhydroxyalkanoate copolymers and evaluation of their blends by fourier transform infrared spectroscopy and scanning electron microscopy. Indian J. Microbiol..

[28] Fu L., Hsu J., Shih M., Hsieh C., Ju W., Chen Y., Lee B., Hou C. (2021). Process Optimization of Silver Nanoparticle Synthesis and Its Application in Mercury Detection. Micromachines.

[29] Talabani R.F., Hamad S.M., Barzinjy A.A., Demir U. (2021). Biosynthesis of Silver Nanoparticles and Their Applications in Harvesting Sunlight for Solar Thermal Generation. Nanomaterials.

[30] Elzey S., Grassian V.H. (2010). Agglomeration isolation dissolution of commercially manufactured silver nanoparticles in aqueous environments. J. Nanopart. Res..

[31] Schuster D. (1987). Encyclopedia of Emulsion Technology, Vol. 3: Basic Theory, Measurement, Applications.

[32] Hansen P.E., Vakili M., Kamounah F.S., Spanget-Larsen J. (2021). NH Stretching Frequencies of Intramolecularly Hydrogen-Bonded Systems: An Experimental and Theoretical Study. Molecules.

[33] Gumel A.M., Annuar M.S.M., Heidelberg T. (2012). Biosynthesis and Characterization of Polyhydroxyalkanoates Copolymers Produced by Pseudomonas putida Bet001 Isolated from Palm Oil Mill Effluent. PLoS ONE.

[34] Kalaoglu-Altan O.I., Baskan H., Meireman T., Basnett P., Azimi B., Fusco A., Funel N., Donnarumma G., Lazzeri A., Roy I. (2021). Silver nanoparticle-coated polyhydroxyalkanoate based electrospun fibers for wound dressing applications. Materials.

[35] Feng Q.L., Wu J., Chen G.Q., Cui F.Z., Kim T.N., Kim J.O. (2000). A mechanistic study of the antibacterial effect of silver ions on *Escherichia coli* and *Staphylococcus aureus*. J. Biomed. Mat. Res..

[36] Jung W.K., Koo H.C., Kim K.W., Shin S., Kim S.H., Park Y.H. (2008). Antibacterial activity and mechanism of action of the silver ion in *Staphylococcus aureus* and *Escherichia coli*. Appl. Environ. Microb..

[37] Song H.Y., Ko K.K., Oh L.H., Lee B.T. (2006). Fabrication of silver nanoparticles and their antimicrobial mechanisms. Eur. Cells Mater..

[38] Kaviya S., Santhanalakshmi J., Viswanathan B. (2011). Green synthesis of silver nanoparticles using *Polyalthia longifolia* leaf extract along with D-sorbitol: Study of antibacterial activity. J. Nanotech..

[39] Shrivastava S., Bera T., Roy A., Singh G., Ramachandrarao P., Dash D. (2007). Characterization of enhanced antibacterial effects of novel silver nanoparticles. Nanotechnology.

[40] Rodrigues A.M., Guardão Franca R.D., Dionísio M., Sevrin C., Grandfils C., Reis M.A.M., Laorenço N.D. (2022). Polyhydrox-yalkanoates from a Mixed Microbial Culture: Extraction Optimization and Polymer Characterization. Polymers.

[41] Mahato R.P., Kumar S., Singh P. (2021). Optimization of Growth Conditions to Produce Sustainable Polyhydroxyalkanoate Bioplastic by *Pseudomonas aeruginosa* EO1. Front. Microbiol..

[42] Rampado R., Giordano F., Moracci L., Crotti S., Caliceti P., Agosti M., Taraballi F. (2022). Optimization of a detergent-based protocol for membrane proteins purification from mammalian cells. J. Pharm. Biomed. Anal..

[43] Lemoigne M. (1926). Produits de deshydration et de polymerisation de L’acide = Oxybutyrique. Bull. Soc. Chim. Biol..

[44] Law J.H., Slepecky R.A. (1961). Assay of poly-beta-hydroxybutyric acid. J. Bacteriol..

[45] Khatoon U.T., Velidandi A., Nageswara Rao G.V.S. (2023). Sodium borohydride mediated synthesis of nano-sized silver particles: Their characterization, anti-microbial and cytotoxicity studies. Mater. Chem. Phys..

[46] Balcucho J., Narváez D.M., Tarazona N.A., Castro-Mayorga J.L. (2023). Microbially Synthesized Polymer-Metal Nanoparticles Composites as Promising Wound Dressings to Overcome Methicillin-Resistance Staphylococcus aureus Infections. Polymers.

[47] Potter M., Steinbüchel A. (2005). Poly (3-hydroxybutyrate) Granule-Associated Proteins: Impacts on Poly (3-hydroxybutyrate) Synthesis and Degradatio. Biomacromolecules.

[48] Frone A.N., Nicolae C.A., Eremia M.C., Tofan V., Ghiurea M., Chiulan I., Radu E., Damian C.M., Panaitescu D.M. (2020). Low molecular weight and polymeric modifiers as toughening agents in poly (3-hydroxybutyrate) films. Polymers.

[49] Sedlacek P., Pernicova I., Novackova I., Kourilova X., Kalina M., Kovalcik A., Koller M., Nebesarova J., Krzyzanek V., Hrubanova K. (2020). Introducing the Newly Isolated Bacterium Aneurinibacillus sp. H1 as an Auspicious Thermophilic Producer of Various Polyhydroxyalkanoates (PHA) Copolymers–2. Material Study on the Produced Copolymers. Polymers.

