# Supplementary material for: Biosynthesis of Polyhydroalkanoates Doped with Silver Nanoparticles Using Pseudomonas putida and Pseudomonas aeruginosa for Antibacterial Polymer Applications
Source: Int J Mol Sci. 2024 Aug 19;25(16):8996. doi: 10.3390/ijms25168996 (PMC11354355; doi:10.3390/ijms25168996)
Supplement: Supplementary file 1 [file ijms-25-08996-s001.zip › ijms-3138784-supplementary.pdf]

Supplementary information

The XRD analysis is included in the supplementary information. The XRD spectrum of PHAs-Gly exhibited broad bands around 2θ = 20.10°, 30.24°, and 39.91° (Figure S1). The peak near 20.10°, as well as the hump at 30.26°, can be attributed to the XRD pattern of poly(3-hydroxybutyrate) (P(3HB)) according to the literatura[48]. On the other hand, the shifted signals may be due to some residual moisture in the material, while the broad bands are likely due to the contribution of both the crystalline peak of mcl-PHA and the amorphous phase of P(3HB) described in the literature [49].

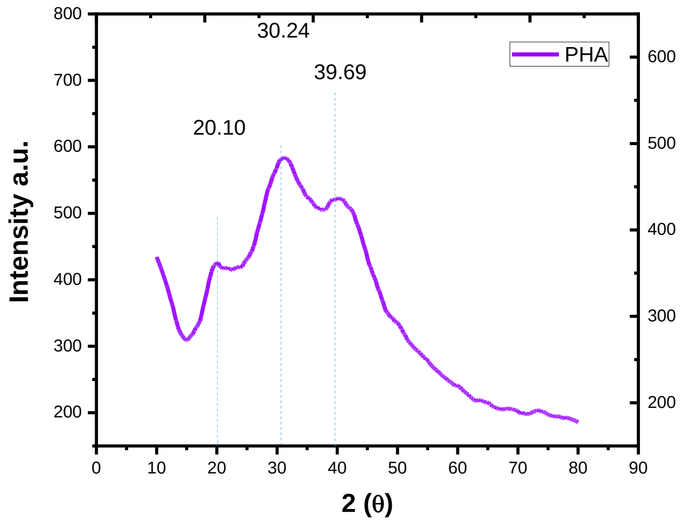

Figure S1. X-ray diffraction pattern of PHAs obtained from *P. aeruginosa* strain and reagent-grade glycerol.
